# Commensal resilience: ancient ecological lessons for the modern microbiota

**DOI:** 10.1128/iai.00502-24

**Published:** 2025-05-19

**Authors:** Abigail E. Rose, Ryan T. Fansler, Wenhan Zhu

**Affiliations:** 1 Department of Pathology, Microbiology, and Immunology, Vanderbilt University Medical Center 204907, Nashville, Tennessee, USA; 2 Vanderbilt Institute for Infection, Immunology, and Inflammation, Vanderbilt University Medical Center 12328, Nashville, Tennessee, USA; University of California at Santa Cruz Department of Microbiology and Environmental Toxicology, Santa Cruz, California, USA

**Keywords:** microbiome, resilience, gut inflammation

## Abstract

The gut microbiota constitutes a complex ecosystem essential for host health, offering metabolic support, modulating the immune system, and protecting against pathogens. However, this community faces constant destabilizing challenges, including dietary changes, antibiotics, and enteric infection. Prolonged microbiota imbalance or dysbiosis can exacerbate intestinal disease states, including inflammatory bowel disease and colorectal cancer. Understanding the mechanisms that sustain microbiota resilience in the face of these imbalances is crucial for maintaining host health and developing effective therapeutics. This review explores microbiota resilience through the lens of an ecological model, emphasizing the interplay between microbial communities and host-driven environmental controls. We highlight two critical factors shaping microbiota resilience: oxygen tension and iron availability—challenges encountered by ancient anaerobic organisms during early evolutionary history, from which the predominant members of the microbiota have descended. Disruptions in intestinal anaerobiosis during inflammation increase luminal oxygen levels, favoring pro-inflammatory facultative anaerobes and depleting obligately anaerobic commensals. Simultaneously, host nutritional immunity restricts iron availability, further challenging commensal survival. This dual environmental challenge of rising oxygen tension and reduced iron availability is a convergent outcome of a diverse array of perturbations, from pathogen invasion to antibiotic treatment. By highlighting these conserved downstream environmental challenges rather than the specific upstream perturbations, this ecological view offers a focused framework for understanding microbiota resilience. This perspective not only enhances our understanding of host-microbiota interactions but also informs therapeutic strategies to foster resilience and support host health.

## INTRODUCTION

Life on Earth emerged around 3.8 billion years ago, in the absence of molecular oxygen (O_2_) (1). At that time, iron, in its soluble ferrous (Fe(II)) form, was the most abundant transition metal in the primeval ocean (2). Its versatility in catalyzing redox reactions and its high bioavailability drove the evolution of iron-based catalysis in early microbial life forms (3). Moreover, the absence of molecular oxygen also permitted radical-based chemistry (4), which, together with iron-based catalysis, powered a broad range of essential metabolic pathways in anaerobic organisms, including those in central metabolism, amino acid metabolism, and the production of DNA (5). Consequently, anaerobic microorganisms, which cannot grow in the presence of oxygen, dominated the blue planet for nearly 1.3 billion years.

This dominance began to wane with the emergence of *cyanobacteria*, which spurred the initial accumulation of oxygen on Earth through oxygen-evolving photosynthesis (6). The resulting oxygen gradually reacted with and depleted reductants, such as sulfur and ferrous iron species in the ocean. Over time, molecular oxygen accumulated in the atmosphere, leading to what is known as the Great Oxidation Event (GOE) (6). This fundamental shift in Earth’s chemistry decreased the bioavailability of iron, a once readily accessible catalyst, by oxidizing soluble ferrous iron (Fe(II)) into insoluble ferric iron (Fe(III)). Coupled with the lethal challenge posed by oxygen itself, these conditions drove the massive extinction of the anaerobic organisms that once dominated Earth (7).

While some organisms evolved to harness O_2_ for metabolic gain, modern descendants of ancestral anaerobes dwell in anoxic habitats, such as the ocean floor, sediment, and the mammalian intestine (8). Over the past few decades, the anaerobe populations in the mammalian gut have garnered significant scientific interest. Together with other microbes, they form a complex microbial ecosystem known as the gut microbiota (9). At homeostasis, this community provides pivotal functions to host health, including aiding host metabolism (10, 11), immune system development (12, 13), and protection against invading enteric pathogens via niche preemption and colonization resistance (14, 15). However, similar to other ecosystems, the gut microbial community faces constant perturbations, including rapidly changing diets (16), antibiotic treatments (17), and invasion by enteric pathogens (18). These diverse perturbations often converge into conserved environmental changes in the gut, such as increased oxygenation and decreased iron bioavailability, the same challenges faced by the ancestors of the gut microbiota members billions of years ago. These environmental challenges drive transient shifts in microbiota composition and function. As an ecosystem, the commensal microbiota may persist in equilibrium during the perturbation or rapidly recover once it subsides, a phenomenon collectively referred to as resilience (19). When perturbation overcomes resilience, the microbiota shifts to an imbalanced state known as dysbiosis (20). In susceptible individuals, a dysbiotic microbiota can aggravate a broad range of inflammatory disease states, including inflammatory bowel disease (IBD), colorectal cancer (CRC), and enteric infections (18, 21, 22).

By preserving microbiota structure and function in the face of perturbations, microbiota resilience serves as a cornerstone for maintaining long-term stability in host-commensal relationships and overall host health. Unraveling the mechanisms underpinning this resilience offers a promising avenue for developing therapeutic strategies to mitigate various intestinal disorders. However, our limited knowledge of microbiota resilience represents a significant gap in our broader scientific understanding of microbial ecology and poses a challenge to designing successful interventions. Thus, a deeper understanding of the intestinal microbiota not only stands to enhance our grasp of host-microbiota interactions and their impact on human health but will also guide the development of more effective and precise treatments.

## DEFINING MICROBIOTA RESILIENCE: INSIGHTS FROM THE ECOLOGICAL MODEL

Discerning the mechanisms of microbiota resilience is complicated by the difficulty in defining what constitutes a healthy microbiome (23). While advances in high-throughput sequencing have aided in furthering our understanding of the microbiota, they have also revealed the immense complexity of this community (24). Each individual’s gut microbiome comprises a unique community of microbial species, many of which resist culture-based characterization, exhibiting constant fluctuations in genetic regulation and metabolism (25). Such complexity complicates the long-sought goal of defining a healthy microbiome based on species composition. The scope of this microbial diversity is matched by a dizzying array of stressors that perturb the microbiota community, including dietary changes, enteric infection, and antibiotic treatment (17, 26). Therefore, identifying a set of core mechanisms for resilience consistent across highly variable microbiotas and a myriad of potential stressors becomes a daunting task.

**Fig 1:**
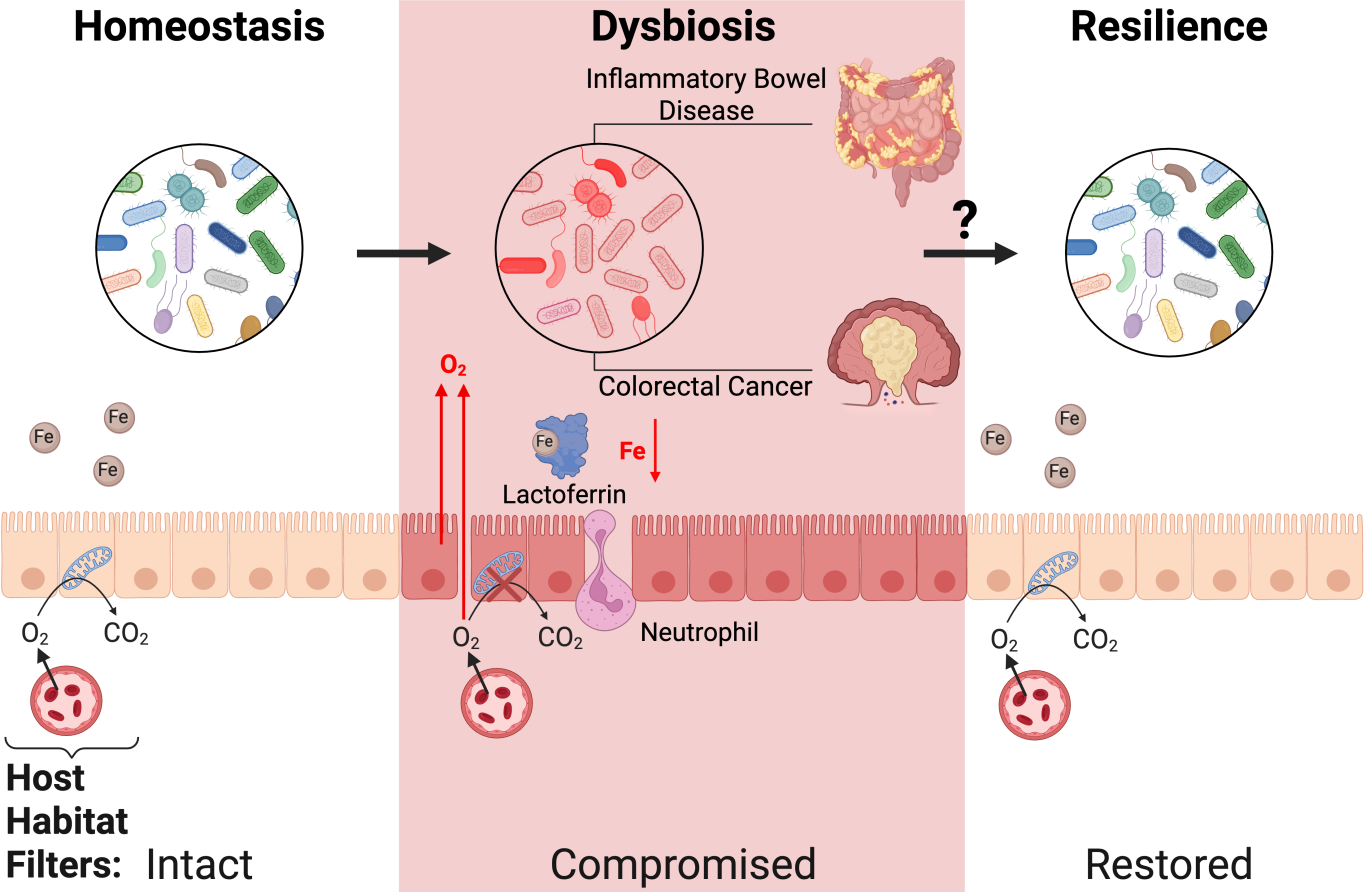
During homeostasis, host-driven habitat filters, such as the maintenance of anoxia by epithelia β-oxidation and stable iron levels through dietary intake, curate a diverse microbiota dominated by beneficial obligate anaerobic primary fermenters. Upon onset of diverse inflammatory intestinal disease states, including inflammatory bowel disease and colorectal cancer, host habitat filters become compromised. Inflammation-driven increases in luminal oxygen levels favor facultatively anaerobic pathobionts at the expense of commensal obligate anaerobes. In parallel, host nutritional immunity factors, such as lactoferrin, sequester iron to defend against potential bacterial pathogens. These environmental perturbations can drive the microbiota to an imbalanced, pro-inflammatory dysbiosis. However, a resilient microbiota can withstand or rapidly recover from these perturbations until host habitat filters are restored. The mechanisms that mediate this resilience and restoration of host habitat filters remain an active area of research (see Fig. 2 to 4).

In response to the difficulty in defining a healthy microbiota solely based on species composition, a new ecological framework to understand the microbiome has gained prominence in recent years. Rather than viewing the microbiome as just the microbial community, this emerging paradigm reconsiders the microbiome as the dynamic interactions between the microbiota and its environment within the host (27–29). In this model, the host curates its resident microbiota via habitat filters, such as controlling the availability of respiratory electron acceptors (20, 30). A healthy microbiome is, therefore, defined by intact host-driven environmental control, while dysbiosis occurs when these functions fail (Fig. 1). The strength of this model is its simplicity. Instead of attempting to reconcile vast omics datasets, this approach focuses on a much smaller list of host functions that shape the gut microbial ecosystem. The individual microbial species “trees” are looked past to see the “forest,” a homeostatic ecosystem maintained by the host.

This ecological view of the microbiome offers a crucial roadmap for elucidating commensal resilience mechanisms. Innumerable interrogations into how compositionally variable microbiotas respond to every potential perturbation can instead be replaced with the more manageable question of how the commensal microbiota adapts to failures in host habitat filtering (resilience vs. dysbiosis) (Fig. 1). One of the most crucial host habitat filters is the maintenance of anaerobiosis within the intestinal lumen via the high mitochondrial consumption of oxygen by colonocytes operating β-oxidation (31). This filter ensures the dominance of obligate anaerobes, bacteria that fail to grow in the presence of oxygen, including *Bacteroides* and *Firmicutes* in the large intestine (32). These fermenters can metabolize the dietary fibers inaccessible to host digestion in the small intestine, releasing short-chain fatty acids (SCFAs) as end products that feed the host via β-oxidation (33). Intestinal inflammation resulting from various perturbations, such as antibiotic use, enteric pathogen infections, non-infectious colitis, and microbially induced tumorigenesis, can lead to alterations in colonocyte metabolism, ultimately causing elevated luminal oxygen levels. This increased intestinal oxygenation depletes obligate anaerobes and fosters the overgrowth of facultative anaerobes, such as *Enterobacteriaceae*—a well-recognized hallmark of dysbiosis (34–44). Thus, the ecological model reveals that a broad array of inflammatory challenges can converge on conserved environmental pressure, such as increased luminal oxygen levels, which any commensal resiliency program must address. Despite the complexity of the microbiota and its potential disruptors, we can reasonably expect that a resilient microbiota, along with effective therapeutic interventions, should be able to manage periods of increased colonic oxygen tension.

The ecological model of the microbiome can uncover additional core resilience factors that come into play as the host attempts to regain control upon the onset of dysbiosis. Iron, a highly versatile cofactor essential for critical cellular functions such as energy generation and DNA replication (45), is a key factor in this process. Given its importance, it is unsurprising that hosts have evolved to restrict iron availability as a habitat filter. Vertebrate hosts exploit the microbial dependency on iron and other nutrient metals to control microbial growth, a process known as nutritional immunity (46). During episodes of gut inflammation, mucosal epithelial cells and immune cells restrict bacterial proliferation by producing iron-scavenging proteins, such as lactoferrin and calprotectin (47, 48). This response leads to depleted luminal iron levels, as observed during enteric infection (49) and CRC (50). Regardless of its precise composition, a resilient microbiota must be capable of acquiring iron in this highly restricted environment. These examples illustrate how the ecological model distills the complexity of the microbiota to discern that commensal resilience strategies must counter increasing oxygen levels and decreased iron availability in the inflamed gut. These represent only two examples of the numerous insights offered by this model. The mechanisms and implications of host habitat filters on the spatial heterogeneity (51) of the intestinal microbiota and host curation of the microbiota composition (52) have been extensively reviewed elsewhere.

## INTESTINAL IRON AVAILABILITY AND ACQUISITION

Under physiological conditions, iron primarily exists in the ferrous (Fe(II)) or ferric (Fe(III)) states (53). The ferrous redox state predominates under anoxic or acidic conditions, while the insoluble ferric state is most prevalent under aerobic conditions (54). While the ferrous state can exist in its free form or in the tetrapyrrole cofactor heme, the ferric counterpart is almost always complexed within iron-chelating factors (55). In either form, iron cannot freely cross biological membranes. Consequently, iron transporters are widespread across phyla, with receptor systems dedicated to the uptake of either complexed ferric iron or free ferrous iron.

Dietary iron comprises heme iron, mostly derived from hemoglobin and myoglobin of animal tissues, and non-heme iron, present in both plant- and animal-based diets. In vertebrates, heme-iron is absorbed much more efficiently than non-heme iron, with approximately 15–25% of dietary heme-iron absorbed in the duodenum compared to 5–10% dietary non-heme iron (56). Iron is tightly sequestered in vertebrate tissues upon absorption to both restrict its acquisition by microbial cells and prevent the toxic production of ROS by the Fenton and Haber-Weiss reactions (57). Intracellular iron is bound within metalloproteins or stored in ferritin, while extracellular iron is complexed as heme in hemoglobin or sequestered by transferrin and calprotectin (46). As discussed above, host iron-chelating proteins serve as an innate defense mechanism, restricting microbial access to iron and protecting against infection. This restriction is further tightened upon inflammation onset. The expression of calprotectin significantly increases during bacterial infection, accounting for up to 50% of the total protein content of neutrophils (58). The combined action of these host chelators results in an exceedingly low free iron concentration within the host (10^−24^ M) (59–61). With access to this essential nutrient under such strict control, the host heme pool is an attractive iron source to pathogenic bacteria. Many genera encode hemolysins to mediate the lysis of erythrocytes and the consequent release of heme and hemoglobin (62). To counteract this, the host employs proteins like hemopexin and haptoglobin to sequester free heme and hemoglobin, respectively (63). This, however, is exploited by the iron-regulated surface determinant (Isd) system, a canonical mechanism for direct heme uptake in Gram-positive bacteria. The surface receptors IsdB and IsdH bind hemoglobin and hemoglobin-haptoglobin, respectively. Upon surface binding, IsdA and IsdC then transport the heme across the cell wall, and the ATP-binding cassette (ABC) transporter IsdDEF shuttles heme into the cytoplasm (47). Gram-negative bacteria can acquire heme through the secretion of heme-binding proteins (hemophores), followed by uptake via TonB-dependent receptors on the outer membrane (64).

While heme is the primary source of iron derived from animal-based diets, ferric iron complexes, including tannins, polyphenols, and phytates, are available from dietary plants (60). The importance of these compounds to microbiota iron acquisition remains unclear, but there is reason to believe they may be a relevant iron source to intestinal microbes. These complexes are not efficiently absorbed by the host, making them more likely to reach the colon (65). Some gut microbes, such as *Bifidobacteriaceae* (66)*,* can digest phytate, as evidenced by the presence of phytate degradation products in the colons of conventional, but not germ-free, rats (60, 67). This may represent a strategy by which to liberate phytate-bound iron. The ferric iron in these complexes may also be accessible to capture by tighter binding siderophores (see below) (68). However, further studies are needed to determine the significance of these plant-derived compounds as an iron source to the gut microbiota.

In addition to complexed ferric iron, bacteria can directly import free ferrous iron. The major ferrous iron uptake mechanism in bacteria is the Feo system. The FeoABC system has been primarily studied in *Enterobacteriaceae,* but homologs exist among several intestinal bacteria phyla (69). FeoB is a GTP-binding membrane protein that transports ferrous iron across the membrane in a mechanism dependent on GTP hydrolysis (70). FeoA is likely a small cytoplasmic protein that may stabilize FeoB and facilitate iron transport (71). FeoC, limited to γ-*Proteobacteria*, is believed to be an Fe-S-dependent transcriptional repressor (72). Feo knockout strains of *Escherichia coli* are incapable of taking up ferrous iron or colonizing the murine gut (73). A fitness dependence on the Feo system for intestinal colonization has also been observed in *Campylobacter jejuni* (74) and *Helicobacter pylori* (75). The Feo system is also present among *Firmicutes*, a prominent phylum encompassing several members of the commensal microbiota (76). Notably, *Firmicutes* also encode extracellular ferric iron reductases involved in extracellular electron transfer (EET). This unique flavin-based EET system shuttles electrons from the cytosol to extracellular iron and allows growth on non-fermentable carbon sources (77). By reducing extracellular ferric iron, this system may provide *Firmicutes* with a localized supply of ferrous iron amenable to Feo-mediated uptake. Together, this evidence suggests that Fe(II) is an important form of this essential metal in the homeostatic gut.

**Fig 2:**
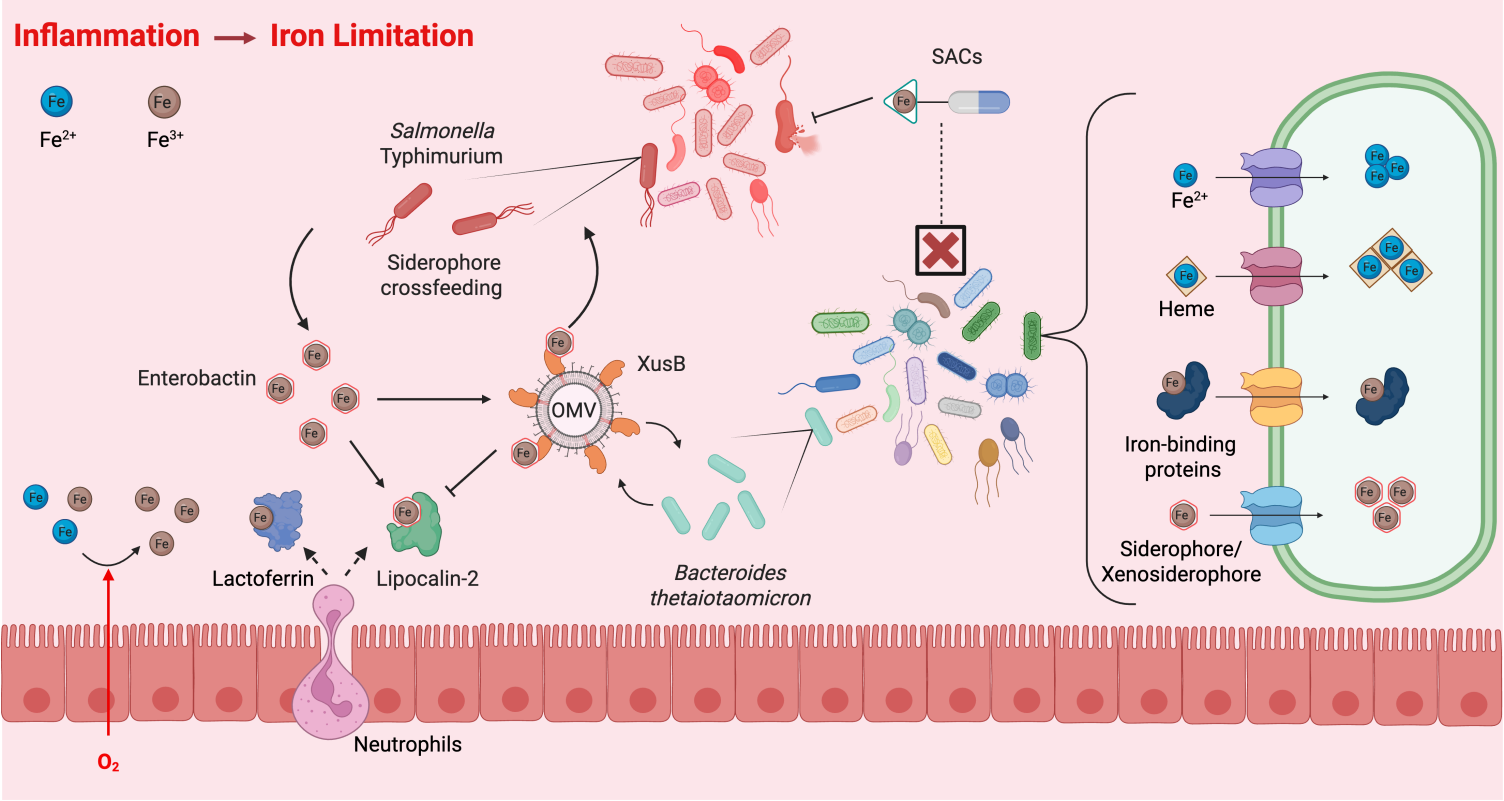
Intestinal bacteria can uptake iron in its free ferrous state or a complexed state in heme, iron-binding proteins, or siderophores. Upon intestinal inflammation, increased luminal oxygen levels can shift redox balance in favor of insoluble ferric iron over soluble ferrous iron. To mitigate pathogen expansion, host nutritional immunity factors, including lactoferrin, sequester iron. Pathogens, such as *Salmonella* typhimurium, produce siderophores, such as enterobactin, to compete with host proteins for iron. In turn, the host secretes lipocalin-2 to sequester enterobactin-bound iron. The prominent intestinal commensal *Bacteroides thetaiotaomicron* cannot produce its own siderophores but can pirate enterobactin via the lipoprotein XusB secreted on outer membrane vesicles (OMVs). While XusB-bound enterobactin is shielded from lipocalin-2, it remains accessible to *S*. Tm. By re-acquiring XusB-bound enterobactin, *S*. Tm exploits commensal iron acquisition to recover a pool of siderophores that are otherwise sequestered by host nutritional immunity. Moreover, certain species of the breakdown products of enterobactin/salmochelin are accessible to *S*. Tm but not commensals, such as *B. theta*. This specificity of siderophore uptake systems offers a means of targeting specific pathogens while sparing the commensal microbiota via siderophore-antibiotic conjugates (SACs).

Large amounts of excess dietary non-heme iron not absorbed in the duodenum would result in a possible iron-replete environment in the intestinal lumen. However, the increasing pH from the duodenum through the small intestine favors the insoluble ferric state, decreasing the bioavailability of iron as it enters the colon (56). During intestinal inflammation, leakage of oxygen into the gut lumen would further shift the redox balance in favor of the insoluble ferric state. Combined with the induction of host nutritional immunity, this shift results in a comprehensive restriction of iron availability to the colon’s microbial inhabitants (Fig. 2). Just as in the aftermath of the GOE, intestinal bacteria require additional tools to survive in this further iron-restricted environment.

## SIDEROPHORES: A LASTING SOLUTION TO IRON LIMITATION

To acquire iron in environments where this essential micronutrient is limited, many bacteria have evolved to produce siderophores, small molecules with high binding affinity for ferric iron (78). *Cyanobacteria*, which played a pivotal role in the initial accumulation of oxygen on Earth—thereby driving the oxidation of soluble ferrous iron (Fe(II)) to insoluble ferric iron (Fe(III)) (79), are thought to be the first organisms to produce siderophores (80). Indeed, as siderophore biosynthetic clusters are present across *cyanobacteria*, their evolution may predate the GOE, as these microbes adapted to oxygen-rich microenvironments of their own making (81). Billions of years later, siderophores remain an essential component of microbial adaptation to the intestinal ecosystem, especially during periods of inflammation when host nutritional immunity further restricts access to this nutrient. Pathogens, such as *Salmonella enterica* serovar Typhimurium (*S*. Tm), can compete for iron with the host by producing siderophores, including enterobactin (59). This microbial iron capture strategy is countered by the host protein lipocalin-2, which sequesters iron-laden enterobactin (Fe-Ent) (82) (Fig. 2). *S*. Tm, in turn, can produce the “stealth siderophore” salmochelin (83), a glycosylated derivative of enterobactin not recognized by lipocalin-2, to evade nutritional immunity and thrive in the inflamed gut. However, until recently, it was not fully understood how commensal bacteria that do not produce any known siderophores, such as the *Bacteroides*, dominant in the human microbiota, maintain resilience in the face of nutritional immunity. Although *Bacteroides* do not synthesize siderophores themselves, they have evolved mechanisms to exploit those produced by other organisms. In response to the mobilization of the nutritional immunity response following *Salmonella* Typhimurium infection, *Bacteroides thetaiotaomicron*, a representative member of the genus, upregulates an operon known as the xenosiderophore utilization system (Xus) (49). Notably, a component of this operon, XusB, is a lipoprotein secreted on outer membrane vesicles that bind iron-laden enterobactin (84). The XusB-bound Fe-Ent is then transported via XusA, a TonB-dependent transporter, into the periplasm where XusC may serve as a reductase to reduce siderophore-bound Fe(III) to Fe(II) (85), facilitating its release. XusABC contributed to *B. thetaiotaomicron* fitness in murine models of infectious and non-infectious colitis (49). Notably, XusB-bound enterobactin is less accessible to host sequestration by lipocalin-2 but can be readily “re-acquired” by *S*. Tm. Thus, XusB-bound Fe-Ent can serve as a “shielded” siderophore pool for *S*. Tm. These findings indicate that XusB cannot only promote *B. thetaiotaomicron* resilience in the inflamed gut but can also aid *S*. Tm in the evasion of host nutritional immunity, revealing a previously unappreciated role for a commensal bacterium in this struggle between host and pathogen (Fig. 2). Furthermore, XusB is structurally conserved across multiple *Bacteroides* species, indicating that this nutrient acquisition strategy is an important component of commensal resilience. Even among siderophore non-producing species, these small molecules that evolved at the onset of the GOE remain essential to bacterial fitness in iron-limited ecosystems.

## INCREASED OXYGEN LEVELS IN THE INFLAMED GUT: THE MODERN GOE

**Fig 3:**
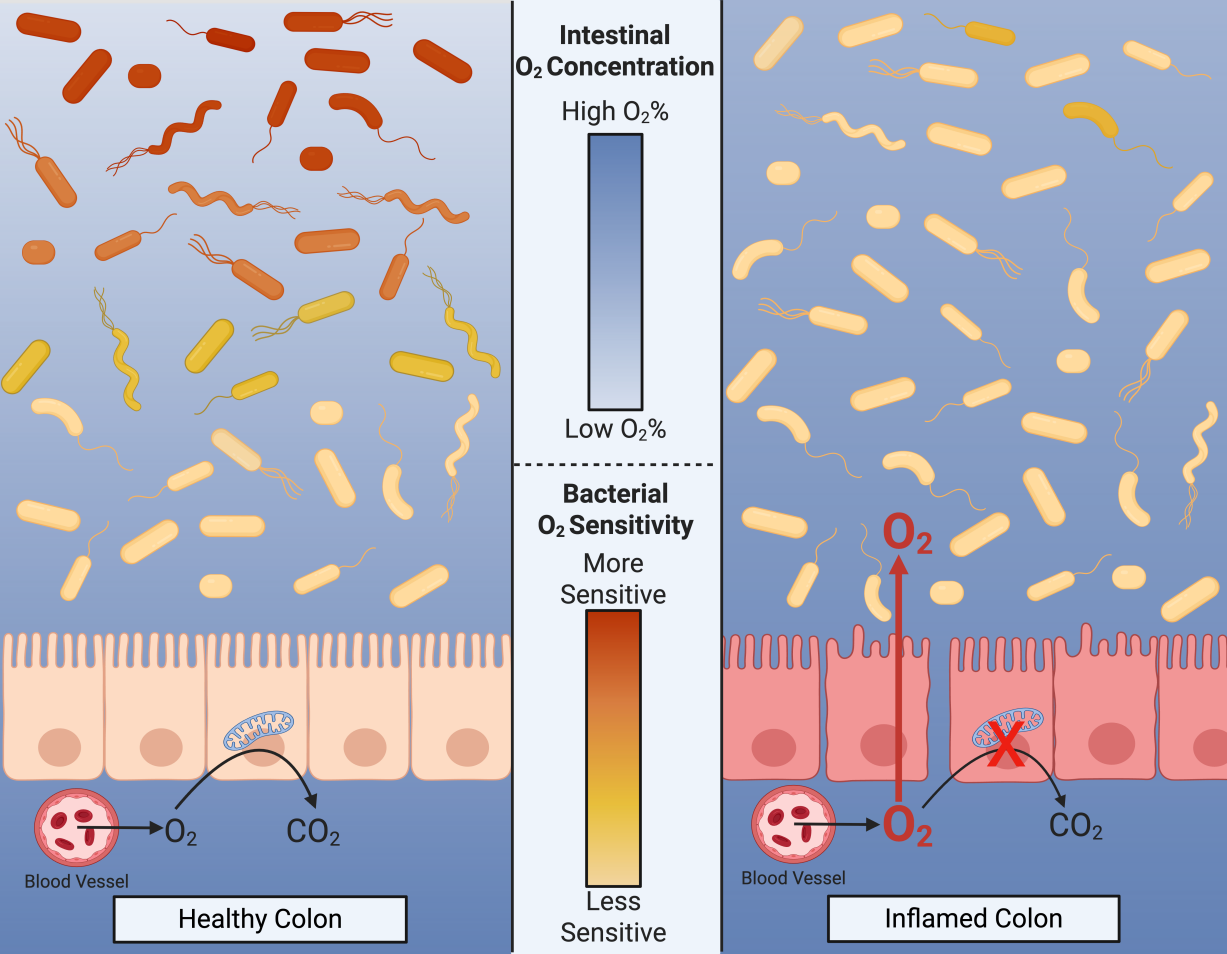
In inflammatory intestinal disease states, the loss of the host-driven habitat filter, such as anoxia maintained by epithelia β-oxidation, results in the depletion of beneficial obligately anaerobic primary fermenters. The loss of commensal anaerobes results in increased nutrient availability in the gut lumen, combined with elevated oxygen levels, driving the expansion of facultative anaerobic pathobionts that can utilize oxygen as an electron acceptor. This shift in microbial composition underscores the profound impact of habitat filter loss on gut microbiota dynamics during inflammation.

Intestinal oxygen tension is another critical habitat filter that governs microbiota composition and function during health and disease (34). Under homeostatic conditions, the unique metabolism in colonocytes establishes a steep oxygen gradient in which colonic oxygen concentrations in the vascularized submucosa are near 7–10% and swiftly drop to approximately 0.4% in the lumen (86–88) (Fig. 3). This oxygen gradient is maintained by several factors, including the oxygen-consuming epithelia β-oxidation, hypoxia-inducible factor (HIF) signaling, and, significantly, the activity of the microbiota itself (86, 89). The oxygen gradient within the gastrointestinal tract dictates the spatial distribution of bacteria, with more aerotolerant and facultative anaerobes occupying regions closer to the epithelium. In contrast, obligate anaerobes thrive in the oxygen-depleted luminal space (90) (Fig. 3).

**Fig 4:**
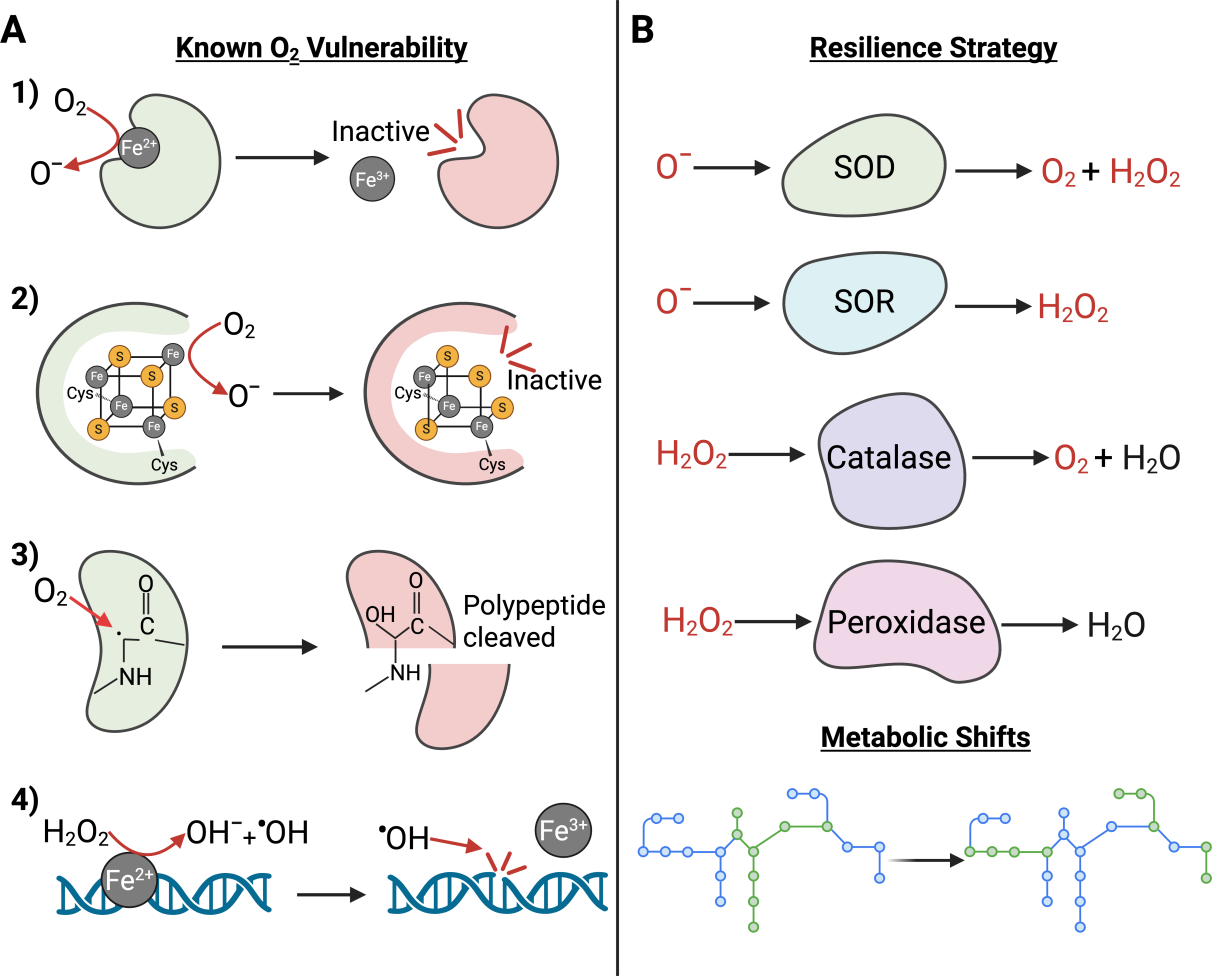
(A) Oxygen thwarts obligately anaerobic bacterial growth by incapacitating essential metabolic functions, which rely on catalytic mechanisms, such as (i) mononuclear iron enzymes, (ii) iron-sulfur clusters, and (iii) glycyl radical enzymes, which are incompatible with molecular oxygen, and (iv) as O_2_ enters the cell, it is quickly reduced by low-redox potential centers, leading to the formation of reactive oxygen species (ROS) that can directly damage DNA. (B) To combat the threat of O_2_, many bacteria produce ROS-detoxifying enzymes, such as catalase, superoxide reductase, and superoxide dismutase, and peroxidases to neutralize the accumulating ROS. Additionally, obligate anaerobes can redirect their metabolism to less oxygen-sensitive pathways or halt ROS-producing metabolic processes until the threat subsides. SOD: superoxide dismutase; SOR: superoxide reductase.

Anaerobes’ sensitivity to oxygen plays a crucial role in shaping microbial niches within the gut. During intestinal inflammation, intestinal epithelial cells' metabolism shifts from oxidative phosphorylation to glycolysis, reducing epithelial oxygen consumption. This metabolic shift weakens the gut’s oxygen barrier (39). Combined with potential disruptions to the physical barrier caused by pathogens (91), oxygen can diffuse more readily into the gut lumen. Oxygen levels of approximately 5% (40 mm Hg) have been observed in healthy colonic tissue and 0.4% (3 mm Hg) in the lumen (87). During intestinal inflammation, this steep oxygen gradient could be disrupted, leading to elevated luminal oxygen levels that may approach tissue oxygen concentrations of approximately 5% (36, 37, 42).

Despite their importance, the mechanisms underlying anaerobes’ sensitivity to oxygen have remained elusive since Louis Pasteur discovered anaerobes over a century ago (92). Recent studies, including those from the Imlay Laboratory, suggest that the key incompatibility of anaerobes with oxygen lies in their “anaerobic excellence”—a term describing the specialized strategies that enable anaerobes to catalyze challenging chemical reactions essential for growth (8, 93). Anaerobes have evolved over billions of years to utilize diverse catalytic strategies, including the use of iron, low-redox potential iron-sulfur clusters, iron-independent low-redox potential centers, or glycyl radical mechanisms to derive energy from growth substrates (8). Low-redox potential centers include moieties that could readily donate electrons to oxygen, such as solvent-exposed Fe(II), reduced iron-sulfur clusters, flavin adenine dinucleotide, and flavin mononucleotide (93, 94). While these strategies are the foundation of anaerobic excellence, they become detrimental in the presence of oxygen (Fig. 4A).

Adventitious collisions between oxygen and low-redox potential chemical groups (e.g., flavin) in microbial cells can drive the reduction of oxygen into ROS, including superoxide and hydrogen peroxide via the Fenton reaction, as well as highly reactive hydroxyl radicals via Haber–Weiss reactions (95). Oxygen can directly oxidize iron in mononuclear iron proteins (Fig. 4A-1) or solvent-exposed iron-sulfur clusters (Fig. 4A-2). In addition to damaging iron centers, oxygen inactivates glycyl radical enzymes by adducting the glycyl radical, forming a hydroperoxyl radical species that, in some cases, cleaves the polypeptide and inactivates the enzyme (Fig. 4A-3) (96, 97). Pioneering work from the Imlay lab and others has demonstrated that these oxidative processes damage a number of essential enzymes utilized by anaerobes through these mechanisms, including the pyruvate splitting enzymes pyruvate formate lyase (PFL), pyruvate: ferredoxin oxidoreductase (PFOR) (98), aconitase (99), and ribonucleotide reductases (RNRs) (100). The oxidation of iron and glycyl radicals within active sites of redox enzymes increases catalytically inactive enzyme pools, compromising essential metabolic pathways and rendering obligately anaerobic bacteria metabolically inert (98). Beyond enzyme inactivation, ROS can wreak havoc in the bacterial cell by causing direct damage to DNA in both the nucleic acid backbone and nucleobases (Fig. 4A–4) (101). In the inflamed gut, this condition is further exacerbated by the robust production of host-derived ROS, a key component of the immune defense during inflammation (102). The combined effects of increased molecular oxygen, heightened ROS levels, and reduced iron availability create lethal conditions for intestinal anaerobes, similar to those faced by their ancient ancestors following the GOE (8). Expectedly, some bacteria have evolved strategies to cope with the dual challenge imposed by molecular oxygen and ROS (Fig. 4).

## SURVIVAL UNDER PRESSURE: COMMENSAL RESILIENCE TO OXIDATIVE STRESS

Anaerobic members of the microbiota may initially attempt to overcome the luminal oxygenation induced by intestinal inflammation by redirecting metabolism to less oxygen-sensitive pathways (103). However, these alternate catabolic routes often yield less energy or generate detrimental secondary metabolites. To mitigate these challenges, many commensal bacteria have evolved specialized mechanisms to directly address the threat of O_2_ and resulting ROS, including reactive oxygen species detoxification and activation of oxidative damage repair mechanisms (Fig. 4B). Furthermore, these responses and the capacity to detoxify and neutralize oxygen and ROS vary significantly among species, resulting in an array of oxygen tolerance and susceptibility amongst commensal organisms, which reflects the oxygen tension present in their specific intestinal niches. As the gut microbiota is comprised of a complex ecosystem characterized by immense diversity, we will highlight what is currently known about the oxidative stress response in representative members of the most abundant bacterial phyla in the mammalian microbiota, *Firmicutes* and *Bacteroidetes*, which make up 90% of the gut microbiota (104).

The genus *Clostridium* constitutes 95% of the *Firmicutes* phylum (104). *Clostridia* are known to contribute to host health in many ways, including the production of SCFAs such as butyrate (105). Butyrate not only serves as the primary energy source for colonocytes (106) but also plays an anti-inflammatory role in the gastrointestinal tract by blocking the expression of pro-inflammatory transcription factors (107) and the production of inflammatory cytokines (107, 108). *Clostridia* are characterized by their strict anaerobic lifestyle; however, recent work has unveiled that many of these bacteria are better equipped to deal with O_2_ than previously thought. Although some *Clostridia* are known to persist in the presence of O_2_ by forming spores, vegetative *Clostridia* have been found to cope with oxidative stress by scavenging and detoxifying O_2_ and implementing repair mechanisms to address macromolecule damage following transient O_2_ exposure (109). One of the most abundant human colonic bacteria, *Faecalibacterium prausnitzii,* is a proficient butyrate-producing strict anaerobe belonging to the class *Clostridia*. However, this bacterium is capable of colonizing regions of the colon with high oxygen tensions via a unique mechanism (110). It was found that this strict anaerobe tolerates periods of high oxygen tension by using an extracellular electron shuttle, which oxidizes reduced electron carriers by transferring electrons to flavins and subsequently reducing O_2_ to harmless water (110). Other mechanisms *Clostridia* employ to neutralize ROS include the expression of genes encoding for ROS scavenging enzymes such as superoxide reductases (SOR) and rubrerythrin (Rbr) (Fig. 4B) (111). SORs detoxify superoxide by directly reducing it to H_2_O_2_, using electrons from electron donors like NADH and other reduced substrates (112). Peroxidases, including the rubrerythrin family proteins, further detoxify the H_2_O_2_ by reducing it to harmless water (112). Notably, these enzymes are effective at scavenging ROS under anaerobic conditions; however, during high levels of oxidative stress, reduced electron donors may eventually deplete, rendering SOR and Rbr ineffective. In more aerotolerant *Clostridia* species, additional scavenging enzymes, such as catalase for H_2_O_2_ detoxification and superoxide dismutase (SOD) for superoxide reduction, have been identified (Fig. 4B). However, these enzymes are infrequently found amongst other *Clostridia* species, and when present, are typically expressed at low levels, consistent with the high degree of oxygen sensitivity in many of these species (113).

*Bacteroides*, in comparison to *Clostridia*, display a wider range of oxygen tolerance, with many of these obligate anaerobes exhibiting some degree of aerotolerance (114). *Bacteroides*, the predominant genus of the phylum *Bacteroidetes*, accounts for approximately 30% of all fecal isolates (115). Abundant species such as *B. thetaiotaomicron* have been considered to alleviate colon inflammation in preclinical models of Crohn’s disease (116), reinforce the intestinal mucosal barrier (117), modulate nutrient metabolism (118), and maintain immune response homeostasis (114). *B. thetaiotaomicron* stands out as an abundant and stable member of the microbiota (119). This stability is primarily attributed to its marked metabolic flexibility: it possesses an extensive repertoire of enzymes that extract nutrients from the environment (120). Additionally, the ability to cope with stints of iron limitation, as previously described above, and withstand limited increases in oxygen tension in the face of inflammation also enhances its resilience and success as a commensal organism in the gut. Remarkably, some *Bacteroides* species employ robust defenses against oxidative stress. These include peroxidases, catalase, SOD, alkyl hydroperoxide reductase, and cytochrome c peroxidase, which collectively mitigate the damaging effects of H_2_O_2_ and other ROS (Fig. 4B) (121, 122). The induction of this consortium of oxidative stress response enzymes and the induction of genes involved in nucleic acid and protein repair following exposure to either H_2_O_2_ or O_2_ are orchestrated by the redox-sensing transcription factor OxyR (123). It is important to note that, despite possessing such an impressive array of oxidative defense, many *Bacteroidetes,* including *B. thetaiotaomicron,* are not able to grow in the presence of oxygen levels exceeding the micromolar range (>~0.5% O_2_) (8). While some of the oxygen vulnerabilities in the key metabolic pathways have been characterized, our understanding of the mechanisms underlying the oxygen sensitivity of these anaerobes remains limited. This underscores the need for future research to comprehensively identify oxygen/ROS-damaged enzymes and establish a causal link between such damage and the inability of these organisms to grow in oxygen-rich environments.

Unlike *Bacteroides* and *Clostridia*, *Lactobacilli* are facultative anaerobes capable of growing in the presence or absence of oxygen while primarily relying on fermentation for energy production (124). Although *Lactobacilli* make up a small percentage of the total bacterial population in the distal gut microbiome, they are abundant in the small intestine, where there is an abundance of amino acids and simple sugars to fuel their fermentative metabolism (125, 126). Due to their residence in the small intestine, *Lactobacilli* must tolerate higher oxygen levels than obligate anaerobes in the distal colon, as the small intestine has a significantly higher oxygen tension (90). *Lactobacilli* are the most frequently used probiotics, as they have the potential to enhance intestinal barrier integrity, confer protection from pathogens by producing antimicrobial peptides, occupy nutritional niches, and address inflammatory conditions (127). In high-oxygen tension environments, many *Lactobacilli* express a manganese (Mn) containing SOD to neutralize superoxide radicals. In strains lacking SOD, high levels of intracellular Mn can non-enzymatically scavenge superoxide (128). Additional enzymes, such as NADH peroxidases, play a vital role in detoxifying H_2_O_2_, as *Lactobacilli* primarily generate energy via fermentation, of which NADH is a product. As such, using NADH peroxidases to address oxidative stress also frees up a pool of NAD+ to maintain redox balance while fermenting sugars (129). However, whether *Lactobacilli* benefit from increased oxygenation in the inflamed gut remains unclear.

## HAVING YOUR CAKE AND EATING IT TOO: THE OXIDATIVE STRESS RESPONSE OF *ENTEROBACTERIACEAE*

Nearly all members of the family *Enterobacteriaceae* are facultative anaerobes, and many species belonging to this family are critical enteric pathogens or pathobionts, including *E. coli* and *S*. Tm (130). These organisms are minor constituents of the microbiota at homeostasis because, unlike the dominant anaerobes in the *Bacteroidetes* and *Firmicutes* phyla, they lack the molecular machinery to forage complex host-derived or dietary glycans, which are the predominant nutrients in the gut (131). However, the flexible metabolisms and robust oxidative stress responses provide *Enterobacteriaceae* significant advantages over the strict anaerobic members of the gut microbiota when the habitat filter afforded by the oxygen gradient is lost during intestinal inflammation. *Enterobacteriaceae* express at least three distinct sets of terminal oxidases to respire oxygen or even hydrogen peroxides at different levels (132–134). They have also evolved to express various metabolic enzymes that are less sensitive to oxygen through a variety of adaptations, such as alternate metalation of metalloenzymes and sequestration of sensitive catalytic sites to less solvent-exposed locations within the enzyme (135). Unlike anaerobes, such as *Bacteroides*, which rely primarily on the OxyR regulon to respond to oxidative stress, *Enterobacteriaceae* utilize a more complex system involving both the OxyR and SoxRS regulons. OxyR primarily regulates responses to peroxide stress, while SoxRS governs responses to superoxide stress (136). This division of labor reflects an evolutionary adaptation to frequent encounters with oxygen in facultative anaerobes, enabling a more precise and effective response to varying ROS depending on environmental conditions. This specialization, combined with an arsenal of oxygen/ROS detoxifying enzymes that these systems regulate, enables *Enterobacteriaceae* to reap the metabolic benefits of respiring oxygen while keeping intracellular ROS levels at bay (137, 138). By utilizing oxygen as a terminal electron acceptor, *Enterobacteriaceae* can also catabolize the non-fermentable microbiota-derived carbon sources (139–141). Furthermore, the oxygenation associated with inflammation undermines colonization resistance against *Enterobacteriaceae* by depleting obligate anaerobic commensals, which, in turn, creates nutritional niches for *Enterobacteriaceae* (Fig. 3). Together, these processes drive the characteristic expansion of *Enterobacteriaceae* in the gut during inflammatory disease states, highlighting the critical role of habitat filters in preserving microbiota structure and function in both health and disease (36, 142, 143).

## COMMANDEERING HABITAT FILTERS: THERAPEUTIC APPLICATIONS

The recovery of a dysbiotic microbiota following a perturbation is contingent on several factors, including the extent of microbial depletion, the host diet, and the availability of environmental reservoirs (144). Although the gut community may be reseeded by surviving microbes or environmental sources (145, 146), recovery is often incomplete, particularly in patients with chronic intestinal inflammation, whose microbiota composition continues to shift over time (147, 148). Moreover, reseeding from environmental sources (144, 149, 150) carries the risk of introducing microbes with antibiotic resistance genes or pathogenic potential, a concern that is especially pronounced in hospital settings (151–154). These risks underscore the urgent need for therapeutics aimed at both preventing inflammatory dysbiosis and promoting safe, effective microbiota recovery.

Understanding commensal resilience mechanisms as strategies to adapt to failing host habitat filters can inform the design of therapeutics for inflammatory dysbiosis and its underlying causes. These emerging therapeutic interventions can be targeted to subvert the mechanisms by which pathogens subvert host habitat filters. For example, siderophore-antibiotic conjugates (SACs) represent a “Trojan-horse” antibacterial strategy in which an antibiotic moiety is attached to a siderophore recognized by the targeted pathogen (155) (Fig. 2). This approach hijacks the pathogen’s strategy for acquiring iron in the inflamed gut, instead delivering an antibiotic warhead. Such a strategy is already utilized in natural interbacterial warfare. The class IIb subfamily of microcins, antimicrobial peptides produced by members of *Enterobacteriaceae*, undergo posttranslational modification to conjugate salmochelin at its C-terminus (156). Under iron-limiting conditions, bacteria that actively uptake these microcins are subsequently killed (157). As many bacterial species employ their own siderophores, the rational design of synthetic SACs represents a potential avenue to target specific bacterial species or strains (158–162). Such therapeutics could also thereby minimize the collateral damage of broad-spectrum antibiotics (162). Thus, the benefit of such a treatment for dysbiosis would be threefold: clearance of the offending pathogen, avoidance of the dysbiotic aftereffects of antibiotic treatment (e.g., debilitating cycles of *Clostridium difficile* infections [163]), and more effective killing of multidrug-resistant strains, as their inherent uptake via siderophore receptors addresses the problem of outer membrane permeability for drug delivery to ram-negative species (164, 165). A better grasp of the mechanisms of xenosiderophore utilization by gut commensals will further inform the design of future SACs to develop more surgical precision in enteric antibiotics. Cefiderocol, the first Food and Drug Administration-approved synthetic SAC for the treatment of multidrug-resistant urinary tract infections (166), comprises a cephalosporin warhead conjugated to a catecholate moiety. Of note, while this catecholate moiety can be recognized and transported by enteric pathogens and pathobiont Enterobacteriaceae [e.g., *E. coli* and foodborne pathogens, such as *Salmonella enterica* serovar Typhimurium are not accessible to commensal members, such as certain strains of *Bacteroidetes* (unpublished observation)]. This specificity will thus represent a starting step for rationally designed SACs that spare the commensals. Looking ahead, therapeutic strategies to manipulate habitat filters could extend beyond controlling intestinal iron availability to include modulating the oxygen landscape of the gut.

Aminosalicylates (5-ASA) exemplify the therapeutic potential of modulating the oxygen landscape of the gut by enhancing epithelial hypoxia, thereby promoting barrier integrity and supporting the growth of beneficial anaerobic commensals (167, 168). By activating peroxisome proliferator-activated receptor gamma (PPAR-γ) and stabilizing HIF, 5-ASA helps restore the physiological hypoxia disrupted in inflammatory conditions, such as IBD (36, 43, 167, 168), highlighting the value of targeting oxygen tension to address intestinal inflammation and microbiota modulation. Presently, the most common clinical interventions for addressing a dysbiotic microbiome include the use of pre- and pro-biotics, as well as fecal transplants (169). While these approaches hold promise, significant challenges arise when considering the use of beneficial anaerobic commensals (170).

One of the primary obstacles is oxygen exposure, as it remains challenging to maintain an anaerobic environment on a commercial scale. This process demands specialized skills, meticulous techniques, and advanced equipment (171). Additionally, anaerobic probiotics face a series of hurdles during production, storage, and distribution, including oxygen exposure, temperature changes, and pH changes throughout the journey from commercial-scale production to storage and distribution before eventually reaching the harsh environment of the consumer’s gastrointestinal system, in which it must successfully colonize and maintain its functional characteristics to be a successful probiotic (172). These obstacles significantly compromise the viability and functional integrity of probiotics, effectively limiting the application of existing probiotics as an effective therapeutic strategy for addressing dysbiosis, especially in the face of high oxygen tensions resulting from inflammation (173–175). These challenges underscore the need for engineered probiotics rationally designed to overcome hurdles such as oxygen exposure during manufacturing and in the host environment. This innovation could significantly enhance the therapeutic potential of probiotics in managing dysbiosis and illuminate the fundamental biological question of why anaerobes are so sensitive to the very molecule you and I cannot live without.

## CONCLUSION

The resilience of the gut microbiota is a cornerstone of host health, reflecting the ability of this dynamic ecosystem to withstand destabilizing perturbations while maintaining functional equilibrium. By adopting an ecological perspective that frames this resilience as adaptations to compromised host habitat filters, we can better understand the common factors that shape the microbiota across diverse inflammatory disease states (Fig. 1). Key challenges faced by the microbiota during dysbiosis—including increased oxygenation and iron restriction—are strikingly reminiscent of the evolutionary pressures faced by ancestral anaerobes during Earth’s Great Oxidation Event. These conserved environmental stressors provide valuable insight into how the modern gut microbiota might respond and recover under perturbation, as well as fundamental biological questions regarding the oxygen sensitivity of strict anaerobes. Furthermore, they underscore the importance of targeting core resilience mechanisms in therapeutic strategies to restore balance in dysbiotic states.

Looking forward, unraveling the molecular and ecological mechanisms governing microbiota resilience will require multidisciplinary approaches that integrate high-throughput sequencing, metabolomics, host-microbe interaction studies, and ecological modeling. Such research promises to illuminate not only the resilience strategies of commensal microbes but also the broader principles of microbial ecology. Ultimately, this knowledge will guide the development of targeted interventions to maintain gut homeostasis, mitigate inflammation, and enhance human health.
